# Hyaluronic Acid in Biomedical Fields: New Trends from Chemistry to Biomaterial Applications

**DOI:** 10.3390/ijms232214372

**Published:** 2022-11-19

**Authors:** Antonia Di Mola, Maria Rosaria Landi, Antonio Massa, Ugo D’Amora, Vincenzo Guarino

**Affiliations:** 1 Dipartimento di Chimica e Biologia “A. Zambelli”, Università degli Studi di Salerno, Via Giovanni Paolo II, 84084 Fisciano, Italy; 2 Institute of Polymers, Composites and Biomaterials, National Research Council of Italy, Mostra d’Otremare, Pad.20, V.le J.F. Kennedy 54, 80125 Naples, Italy

**Keywords:** hyaluronic acid, chemical and physical modification, crosslinking, additive manufacturing, medical and material engineering applications

## Abstract

The aim of this review is to give an updated perspective about the methods for chemical modifications of hyaluronic acid (HA) toward the development of new applications in medical devices and material engineering. After a brief introduction on chemical, structural and biological features of this important natural polysaccharide, the most important methods for chemical and physical modifications are disclosed, discussing both on the formation of new covalent bonds and the interaction with other natural polysaccharides. These strategies are of paramount importance in the production of new medical devices and materials with improved properties. In particular, the use of HA in the development of new materials by means of additive manufacturing techniques as electro fluid dynamics, i.e., electrospinning for micro to nanofibres, and three-dimensional bioprinting is also discussed.

## 1. Introduction: Chemical–Physical Properties and Roles of Hyaluronic Acid

**Figure 1:**
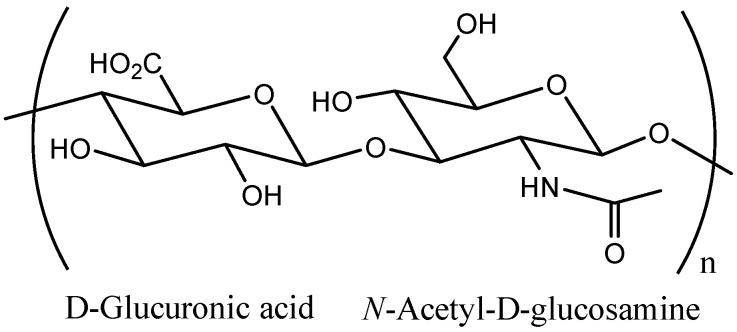
Chemical structure of Hyaluronic acid.

Hyaluronic acid [hyaloid (vitreous) + uronic acid] (HA) is a naturally occurring linear polysaccharide, belonging to the class of non-sulfated glycosaminoglycans (GAGs), with repeating units of D-glucuronic acid and *N*-acetyl-D-glucosamine disaccharide, alternating β-(1→3), β-(1→4) glycosidic bonds (Figure 1) [1]. In 1880, the chemist Portes observed that the mucin in the vitreous body had a different behavior from other mucoids in cornea and cartilage [2]. Later in 1934, Meyer and Palmer isolated a novel GAG from the vitreous of bovine eyes, named HA-based on hyaloid (vitreous) and uronic acid [3].

In this polysaccharide, rotation around the glycosidic bonds is limited because both monosaccharides are in the energetically stable β configuration, in which the bulky functional groups (carboxyl, acetamido, hydroxyl and anomeric carbon) are in the sterically favorable equatorial position [4]. Thus, a rigid conformation is observed whereas hydrophobic CH alternates with polar groups [5], linked by intra- and inter-molecular hydrogen bonds [6]. The number of repeated disaccharides, *n*, in a completed hyaluronan molecule is approximately 10,000 but chains of hyaluronan with up to 25,000 disaccharide units have been reported. HA is a highly hydrophilic polymer that can absorb a large amount of water and expand its solid volume up to 1000 times, forming a loose hydrated network. Since the pKa of HA carboxyl groups is 3–4 [7] and at pH = 7, when these groups are ionized, in vivo, the polymer exists in an ionized form as polyanion (it is generally referred to as hyaluronan) associated with cations (the counterions) which provides structure to tissues by forming viscoelastic hydrogels [8]. Hyaluronan is widespread in all vertebrates in various molecular sizes (M_w_ from 5000 to 20 million); for example, in the umbilical cord, the synovial fluid between joints or in the skin, its concentration is very high (in the human body of an average weight of 70 kg, about 15 g hyaluronan is found in various tissues) [9]. Over 50% of the hyaluronan in the body is present in the skin [10]. Moreover, when Hardingham and Muir found out the interaction of hyaluronan with cartilage proteoglycans, the role of hyaluronan in cellular activity has been highlighted in a large number of publications. For example, it is metabolized in the epidermis and participates actively in many regulatory processes, such as cell proliferation and differentiation [11]. In the dermis, it plays the role of a space-filling material in the extracellular spaces.

The biological properties of HA are size dependent [2]:

(a) High molecular weight (M_w_) HA (M_w_ > 5 × 10^5^) shows space filling, antiangiogenic and immunosuppressive functions;
(b) Medium M_w_ HA (M_w_ between 2 × 10^4^–10^5^) seems to be involved in wound repair, ovulation or embryogenesis;
(c) Oligosaccharides containing 15–50 units (M_w_ between 6 × 10^3^–2 × 10^4^) show inflammatory, immuno-stimulatory and angiogenic actions;
(d) Small M_w_ (M_w_ from 400 to 4000) shows anti-apoptotic effects and can induce heat shock proteins.

Its biological actions are performed based on two mechanisms: it can act as a structural molecule and as a signaling molecule. As mentioned, both of these mechanisms of actions have been highlighted to be size-dependent [2]. High M_w_ HA plays important physiological roles in maintenance of the viscoelasticity, hydration of tissues, water transport and organization of proteoglycans in the extracellular matrix (ECM). HA in the ECM is able to interact with some cell receptors (hyaluronan binding proteins) such as the CD-44, that are involved in cell–cell aggregation, pericellular matrix retention, matrix–cell and cell–matrix signaling [12].

Over the years, HA has found numerous applications in medicine and in cosmetics: it is found in skin creams, eye drops, in products to treat gastroesophageal reflux, in medical devices and material engineering, even in beverage and soft drinks and in soaps. Therefore, the question is spontaneous: is HA miraculous or a false promise? At the point of the state of art it is rather complex to give a comprehensive analysis of all the literature and possible applications of HA. However, the aim of the present work is to provide an overview, given at the same time by a synthetic organic chemist, a material engineer and a medicinal chemist, covering all the main fields of applications with some insights into the development of biomaterials.

## 2. Hyaluronic Acid Modifications

In principle, two different methods can be used to modify HA:

(a) Chemical modifications (crosslinking and conjugation);
(b) Synergistic interaction with other polymers (such as polysaccharides).

### 2.1. Chemical Modifications

HA chemical modifications are mainly performed in aqueous solution involving two sites of the biopolymer: the hydroxyl and the carboxyl groups [13]. Furthermore, the *N*-acetyl group could be modified after the deacetylation, but this is a not common approach due to the possible change in the biological properties of native HA [14]. Actually, the most used solvents for HA chemical modifications are dimethysulfoxide (DMSO) or dimethylformamide (DMF), for reactions in which the reagents are sensitive to hydrolysis [15,16]. However, these approaches require the transformation of HA in a salt soluble in organic solvents by using suitable cations, such as tetrabutylammonium salt [16]. Long final purification processes are also required [14].

**Figure 2:**
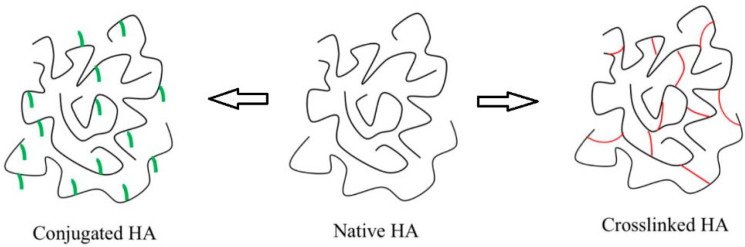
HA forms used for pharmaceutical, medical and cosmetic applications: native, conjugated and crosslinked. Adapted with permission from [13].

The most common techniques for crosslinking and conjugation are based on similar chemical reactions. They differ because conjugation is characterized by grafting a single bond onto an HA chain, while crosslinking means that different HA chains are linked together by two or more bonds [14]. Conjugation and crosslinking are generally carried out for different aims as discussed below, but often strategies to chemically modify HA contain both conjugation and crosslinking processes (Figure 2).

#### 2.1.1. Crosslinking

Chemical crosslinking is a well-known strategy to improve physical–chemical features of native HA as the mechanical, swelling, and rheological properties. Thus, the degradation kinetic of HA-based materials is slowed down and their residence time in the target is enhanced. In particular, covalent crosslinking furnishes the opportunity to obtain hydrogels or cryogels, sponges and other solid forms [17]. Nowadays, several HA-based hydrogels are used in medicine as dermal fillers, viscosupplements and wound dressings, and the market is continuously increasing worldwide. Generally, the most used crosslinkers are small molecules consisting of a spacer and at least two functional groups designed to bridge HA chains. The chemical nature of the crosslinker may also influence gel properties: in fact, a polyfunctional crosslinker may lead to a more difficult degradation [18], while a long and hydrophilic crosslinker backbone may result in undesirably large swelling properties [19].

(a) Crosslinkers for HA’s Hydroxyl Group Modifications

Some common crosslinkers used, for example, to modify HA’s hydroxyl groups are reported in Table 1.

**Scheme 1:**
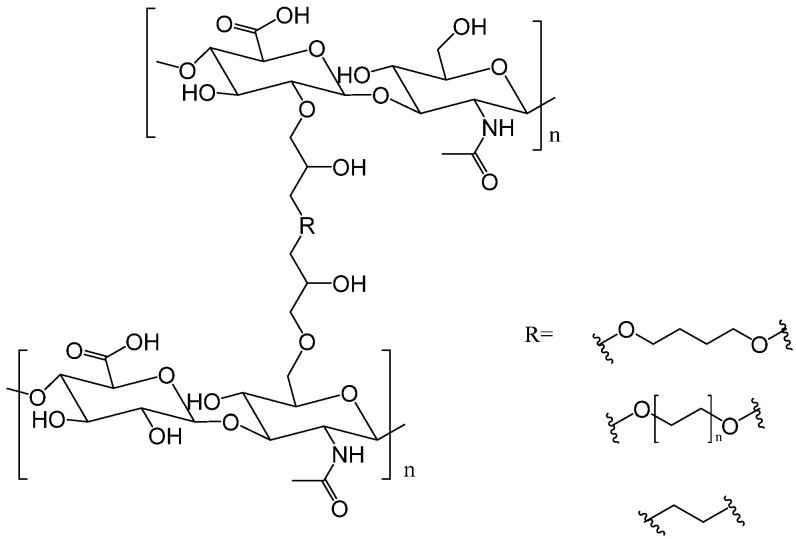
Crosslinking reaction using bisepoxides.

The epoxides act as electrophiles for the Williamson etherification via ring opening (Scheme 1), under strong alkaline conditions [20,21] and the resulting bridge between both HA chains forms an elastic crosslinked HA hydrogel, which has a degree of modification, between 1 and 10% for 1,4-butanediol diglycidal ether (BDDE)-crosslinked HA fillers [20,21]. Restylane, introduced in Europe in 1996 and FDA approved in 2003, is an example of this filler [22].

**Scheme 2:**
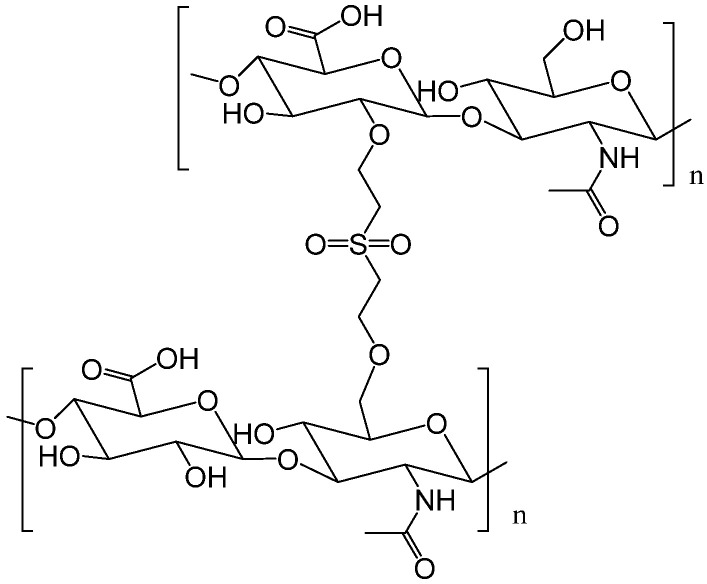
Crosslinking reaction using divinyl sulfone.

Stable three-dimensional hydrogel networks for biomedical applications can also be obtained using divinylsulfone (DVS). It is a bifunctional crosslinker that in alkaline medium forms an ether bond (Scheme 2) with the hydroxyl groups of the HA: Hylaform and Captique were the first DVS based fillers introduced on the US market in 2004 [23].

**Scheme 3:**
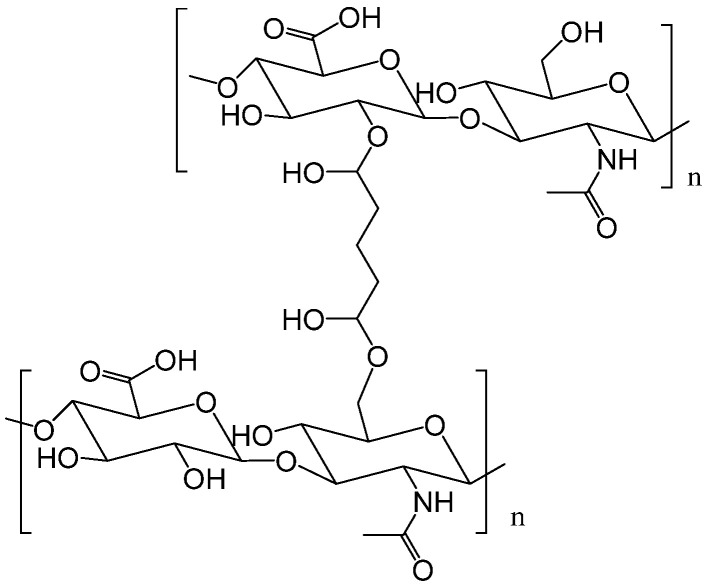
Crosslinking reaction using glutaraldehyde.

Esterification of the hydroxyl groups of HA can also be obtained using octenyl succinic anhydride [24] or methacrylic anhydride [25]. Other bonds, such as hemiacetal bonds, can be formed between the hydroxyl groups of HA and glutaraldehyde (Scheme 3) but, particular attention is necessary during the reaction and purification being glutaraldehyde toxic [14,26,27].

Moreover, the conversion of HA hydroxyl groups to cyanate esters and the subsequent reaction with amines, represents a good strategy to synthesize carbamate derivatives with high degrees of substitution, in only 1 h [28].

(b) Crosslinkers for Hyaluronic Acid’s Carboxyl Group Modifications

Chemical strategies of HA derivatization may also involve esterification and amidation. These reactions can be carried out after the activation of the carboxyl groups by means of different reagents. For example, the transformation of HA carboxyl groups in less hydrophilic esters is a common strategy for decreasing water solubility of HA. This will reduce the susceptibility of HA to degradation, enhancing in situ permanence time [13]. However, HA carboxyl groups are mainly modified through amide formation and some common crosslinkers are reported in Table 2.

**Scheme 4:**
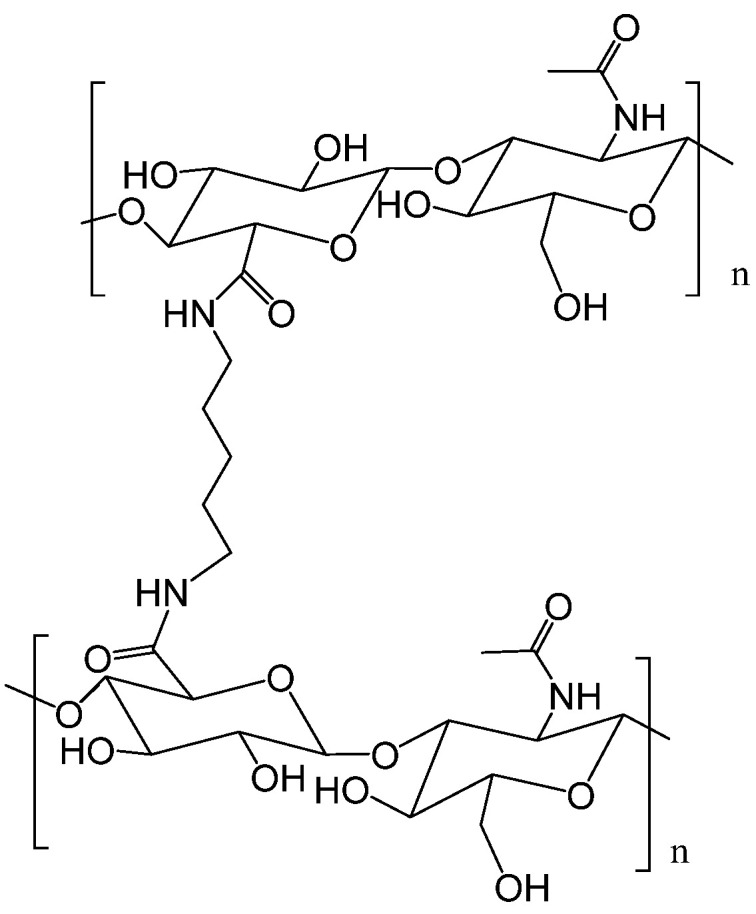
Crosslinking reaction using diamines by Ugi condensation.

Among the developed synthetic procedures, Ugi condensation uses a diamine (Scheme 4), as a crosslinker to form diamide linkages between the polysaccharide chains [14,29].

HA carboxyl groups can be activated by carbodiimide (i.e., N-(3-dimethylaminopropyl)-N-ethylcarbodiimide hydrochloride, EDC) and using co-activators such as N-hydroxysuccinimide (NHS) or 1-hydroxybenzotriazole or triazine compounds. The reaction is suitable also for the derivatization with proteins. Some authors propose carbodiimides also as crosslinker agent. In this way, biscarbodiimides lead to the formation of stable bis(N-acylurea) crosslinked HA-based gel [22,30]. Depending upon the degree of crosslinking, highly swollen gels or virtually insoluble plastic materials can be prepared [31]. Nowadays, one of the most promising strategies for hydrogel preparation appears to be enzymatic crosslinking. This approach is characterized by mild reaction conditions and a fast gelation rate, leading to hydrogels with excellent mechanical properties [32].

#### 2.1.2. Conjugation

This strategy involves a series of chemical transformations, such as the introduction of special functional groups including ether, ester or amide moieties, the attachment of bioactive as well as prodrugs and also the incorporation of marker molecules.

**Scheme 5:**
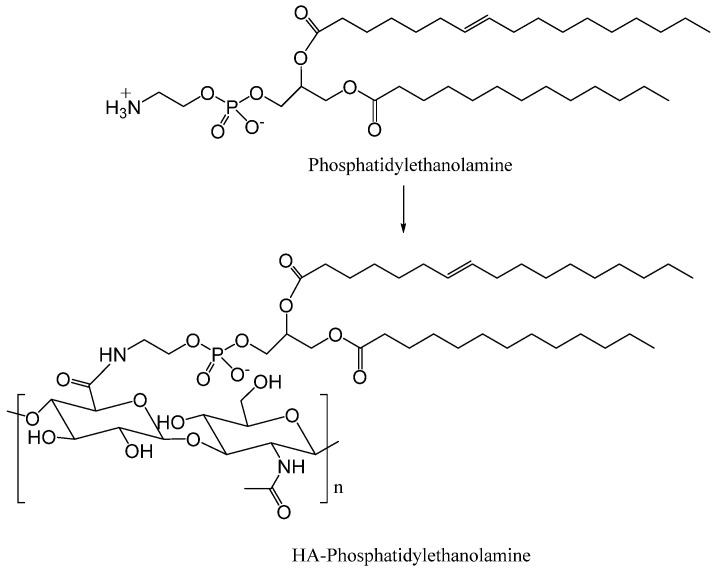
HA conjugation to phosphatidylethanolamine of liposomes.

In particular, HA can be conjugated:

(a) to a carrier system (liposome or nanoparticles) through the amino groups of phosphatidylethanolamine of liposomes (Scheme 5) [33] or through a polylactide-co-glycolide via a polyethylene glycol (PEG) spacer.
(b) to drugs with the aim to improve physical-chemical properties, stability and therapeutic efficacy compared to free drugs.

**Scheme 6:**
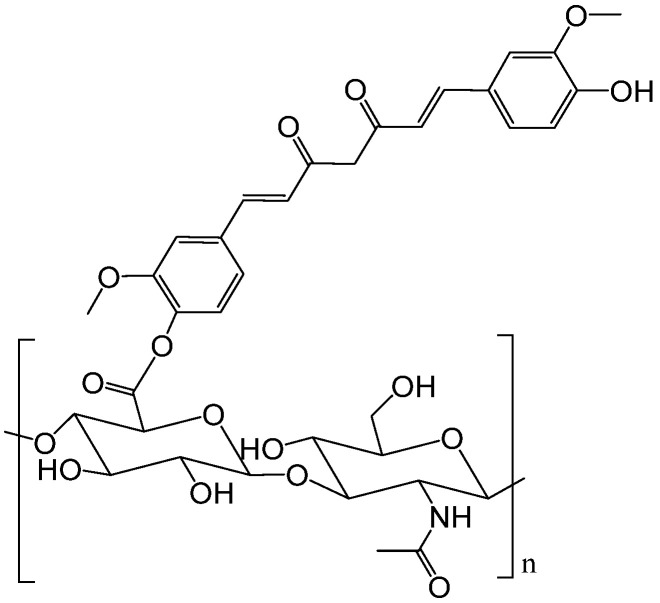
HA–curcumin conjugation via ester linkage.

Conjugation of drugs to HA was first reported in 1991, aiming to produce a pro-drug by the covalent binding of the drug to HA [34,35]. Since HA has a short half-life in the blood, the majority of HA-drug conjugates have been developed for local, i.e., intraarticular, intratumoral, subcutaneous, intravesical and intraperitoneal purposes, rather than systemic administration. Drug–HA conjugates have been prepared with or without the use of any linker. For example, the direct conjugation of curcumin with HA through ester linkage was particularly useful in the enhancement of the stability and water solubility of curcumin (Scheme 6) [36].

Indeed, conjugation of HA with low molecular weight antibacterial agents has provided an effective strategy for developing optimal targeted delivery systems [37,38,39], for example, the amino group of colistin (a polypeptide antibiotic) by carbodiimide chemistry was directly conjugated to the carboxyl groups of HA to form a stable amide bond. The resulted derivative showed slow hydrolysis of amide bond, with a colistin release of 1–5% after 24 h and retained antimicrobial activity [39].

**Scheme 7:**
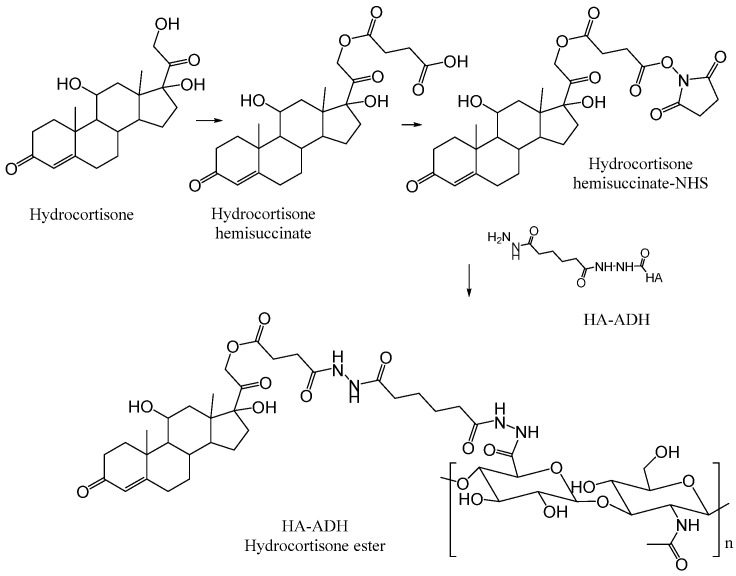
Conjugation onto HA–adipic dihydrazide (ADH) of hydrocortisone hemisuccinate.

Pouyani and Prestwich in 1994 [40] described the grafting of a hydrocortisone, steroidal anti-inflammatory drug, developed for local injections into arthritic joints. HA was first conjugated with adipic dihydrazide (ADH) via its hemisuccinate derivative, which was firstly transformed into an activated ester. In this way, an ester linkage is formed between HA and hydrocortisone that is more easily hydrolyzed by enzymatic mechanisms in the body to release the starting hydrocortisone (Scheme 7).

**Scheme 8:**
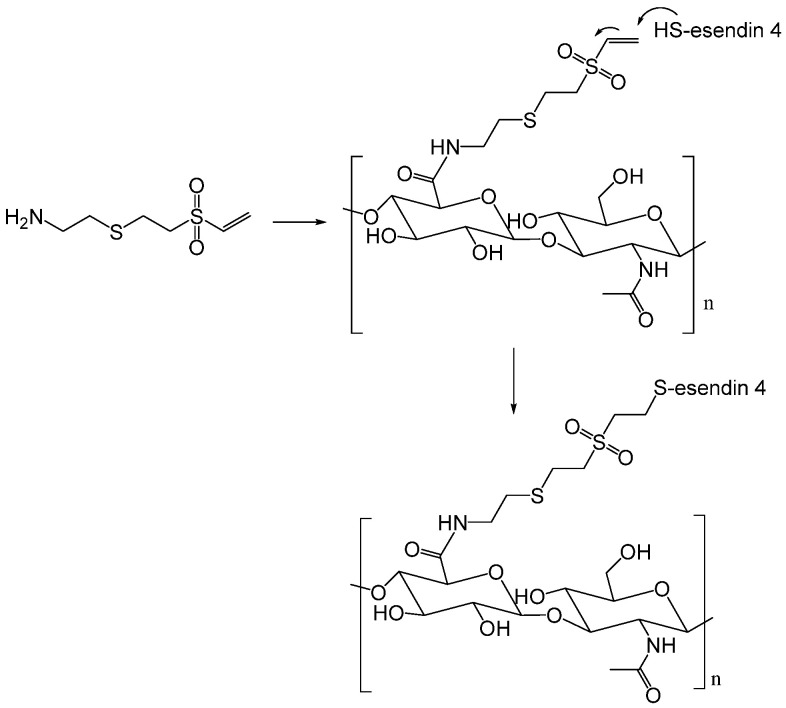
HA–exendin 4 conjugation via vinyl sulfone.

Exendin-4, an antidiabetic peptide, was conjugated to HA via a vinyl sulfone cysteamine intermediate [14,41] and the resulting conjugate showed a longer half-life and better glucose-lowering capabilities than the free drug (Scheme 8).

Another research has shown that conjugation of P40 to HA successfully prevents its systemic absorption, improving its topical therapeutic effect. P40 is a protein fraction isolated from Corynebacterium granulosum (known as Propionibacterium acnes), which shows immunomodulatory, antibacterial, antiviral and antitumor properties [42].

**Scheme 9:**
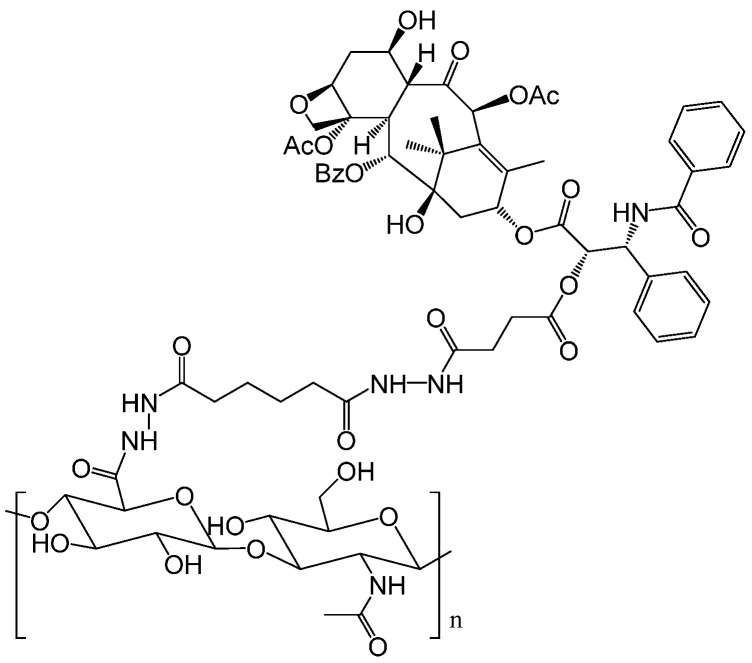
HA–paclitaxel conjugates via N-hydroxysuccinimide (NHS) activated ester.

Conjugations of antitumoral agents to HA have been thoroughly investigated because the active molecules can be strategically delivered to malignant cells by hyaluronan. As mentioned above, hyaluronan is the primary ligand of the CD44 receptor and since it has been shown that the CD44 receptor is overexpressed in a variety of tumor cells, it has been recognized as a powerful tool to develop targeted anticancer therapies [43]. Several works demonstrated that HA can be both a drug carrier and a targeting agent, because of the increase in HA binding and internalization. Paclitaxel was one of the lead compounds studied, because of its poor aqueous solubility. Paclitaxel has been conjugated to HA by several chemical strategies, employing different spacers between HA and paclitaxel, affording derivatives with different chemical linkages and, consequently, with diverse performances. Luo and Prestwich [44], reported modified HA with ADH, followed by the conjugation with a NHS. The ester bond is unstable and by intracellular enzymatic hydrolysis, the drug could be released inside cells. The HA–paclitaxel conjugate is internalized by receptor-mediated endocytosis and only in CD44 overexpressing cells did HA–paclitaxel show a relevant cytotoxicity (Scheme 9).

Different conjugates HA-paclitaxel have been reported [35,45], also using amino acid linkers [46]. With the aim to overcome toxicity and to confer them new physical–chemical properties, such as in Paclitaxel, other anticancer agents were successfully linked to HA [47,48].

### 2.2. Synergistic Interaction

An alternative way for the modification of HA properties, which does not involve chemical derivatization, is the synergistic interaction with other polysaccharides involving molecular association [49]. This is an important phenomenon to study that can be considered a physical crosslinking, because it can produce mixtures with new characteristics and interesting rheological properties with diverse applications; mixtures of polysaccharides can have different properties depending on the ratio of each and the nature of the components [50]. Recently, some works described the behavior of mixtures of HA with different polysaccharides, which are less expensive than HA. The synergism between polysaccharides, besides yielding interesting characteristics and decreasing the cost, also avoids the use of chemicals and further purification processes.

**Figure 3:**
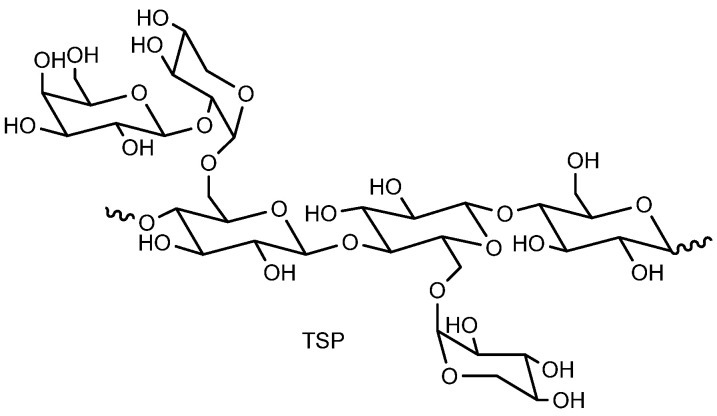
Fragment of TSP (tamarind seed polysaccharide).

In 2010, Barrella et al. described a synergistic interaction between tamarind seed polysaccharide (TSP) and HA in aqueous solution for new formulations for ophthalmic applications with enhanced mucoadhesive properties [51]. Tamarind seed polysaccharide (TSP) is a non-ionic, neutral, branched polysaccharide consisting of a cellulose-like backbone carrying xylose and galactoxylose substituents with a high degree of glycosyl substitution, which is responsible for the formation of stiff extended chain structures in solution (Figure 3) [52,53]. Various TSP/HA mixtures (1/4, 2/3/, 3/2, 4/1) have been studied by Nuclear Magnetic resonance (^1^NMR) spectroscopy and by viscosity measurements, investigating the effect of TSP/HA ratio and total concentration on their capability to form stable aggregates. The experimental data demonstrated that TSP is able to generate inter-chain interactions with HA in TSP/HA non-covalent aggregates and that the minimum concentration of 1.5 mg/mL of each polysaccharide is needed to have the formation of a stable TSP/HA aggregate. In particular, the TSP/HA (3/2) mixture showed a mucoadhesivity stronger than that of each of the unmixed polymers or the less interactive TSP/HA mixtures of different composition, and fulfils the requirements of liquid ophthalmic formulations, such as ocular tolerability and mucoadhesivity, showing relatively low viscosity [54]. Thus, reciprocal synergistic interaction occurs, leading to the basis for the development of a potential excipient for eye drops, composed of a mixture of these polysaccharides.

In 2019, Martin et al. investigated the behavior of mixtures among HA and two galactomannans [55]: guar gum (Cyamopsis tetragonolobo, GG, present in the Leguminosae seeds) and locust bean or carob tree gum (Ceratonia siliqua, LBG).

**Figure 4:**
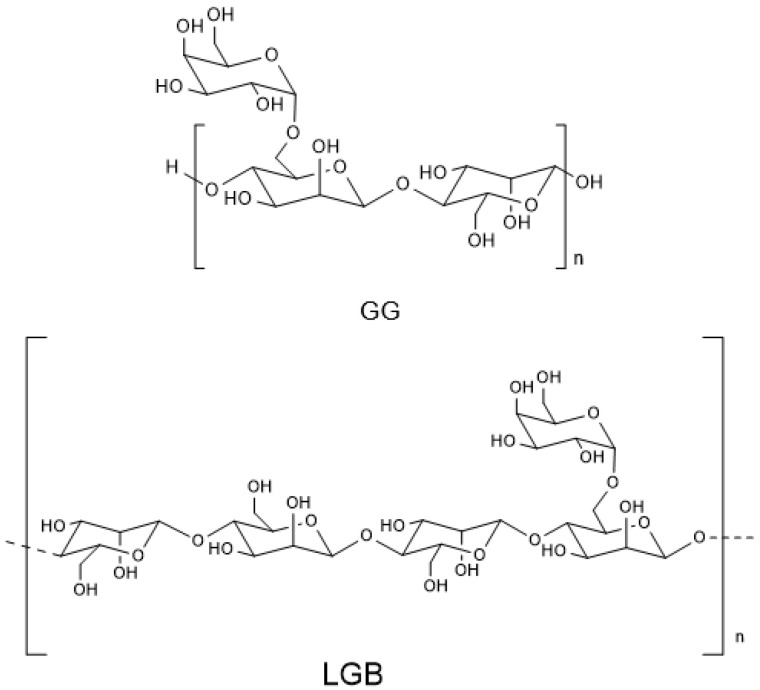
Chemical structure of guar gum (GG) and locust bean gum (LGB).

These galactomannans which are mainly used in food products and cosmetics to modify their viscosity, are formed of a (1 → 4) linked α-D-mannopyranosyl (Man) backbone and substituted by units of β-D-galactopyranose (Gal) (Figure 4) [56]. The substitution’s degree depends on the source and can have several effects on solubility, viscosity and interactions with other polysaccharides. The interactions between these polysaccharides were analyzed by NMR studies and according to rheological properties, the aim is to highlight the behavior of isolated polysaccharides compared to the mixtures. The mixtures containing LGB with 50, 60, 70 and 80% (*w*/*w*) HA showed synergism, while in the case of the HA:GG mixtures synergism was observed in the mixture HA:LBG 80:20 (% *w*/*w*). These results were in according to rheological experiments, demonstrating that the structure of galactomannans is crucial in the interaction with HA (LBG is less substituted by galactose than GG). The hydrogel produced by this synergism (HA: LBG with 50% each (*w*/*w*) showed interesting properties and a great potential for use in nutraceutical foods, tissue engineering and cosmetic products.

**Figure 5:**
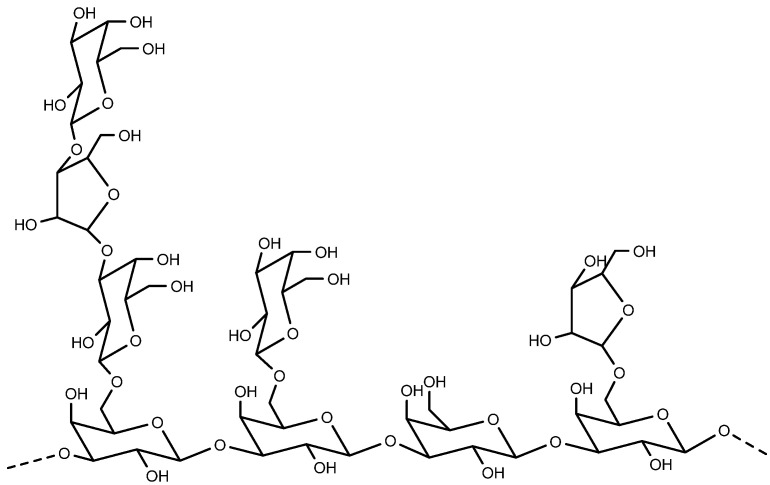
Fragment of Arabinogalactan (AG).

Later, a new artificial drop formulation, containing arabinogalactan (AG) and HA was described, in 2020 by Silvani et al. [57]. AG is a branched polysaccharide, which is usually obtained from the bark of the Larch trees (Figure 5) and it is formed of arabinose and galactose (1:6) and with a small quantity of glucuronic acid [58].

The properties of mixtures of two polysaccharides, AG and HA, were investigated in solution by viscosity measurements and NMR spectroscopy at different AG/HA ratios and concentrations [59]. At the increase in AG concentration, a significant decrease in the viscosity and a concomitant decrease in water protons diffusion coefficients D were highlighted. This can be related to the interesting property that less viscous solutions show progressively reduced mobility of water. In addition, ^1^HNMR investigations demonstrated enhanced affinity of AG/HA mixture at 3/1 ratio toward the molecular probe DS (diclofenac sodium salt) with respect to mucin, highlighting improved muco-adhesive properties of the two polysaccharides mixtures of AG and HA. These mixtures are of high interest because can be useful in the formulation of cosmetics and of new drug release systems, with the purpose to achieve more effective and long-lasting hydration of certain tissues (inflamed skin, dry eye corneal surface, etc.) with the additional advantage of reducing the viscosity of the solutions and enhanced mucoadhesive properties without additional chemical modifications.

As already mentioned, HA is negatively charged polysaccharide and can interact with oppositely charged species to form polyion complexes [60,61]. These complexes can be used in the form of hydrogels, microparticles (as carriers for drug delivery and target therapy), films and scaffolds for tissue engineering [62]. In particular, the electrostatic interactions between HA and oppositely charged polysaccharides lead to self-assembly in aqueous systems as colloidal polyion complex gels or nanoparticles, that possess properties and morphology based on the nature of species involved, solution conditions and the mechanics of assembly [62].

In 2020, Skorik et al. studied the interaction between a water-soluble cationic chitosan derivative (diethylaminoethyl chitosan, DEAE-CS) and HA [63]: chitosan is a cationic polysaccharide that is biocompatible, biodegradable and non-toxic, and it shows biological activity (antimicrobial, antitumor, hypocholesterolemic and regenerative activities). Thus, it is considered an attractive natural polymer for biomedical uses, but under physiological conditions, CS is insoluble in water, so its use is limited. On the other hand, DEAE-CS is a water-soluble cationic chitosan derivative, so its use in the development of polysaccharide-based polyelectrolyte complexes for drug delivery is promising. In their work, the authors demonstrated that mixing of HA with diethylaminoethyl chitosan, DEAE-CS in aqueous solutions is possible to form spherical colloidal nanoparticles. Later, in 2021, the same authors developed colistin (CT)-loaded polymeric carriers based on hyaluronic acid (HA) for potential application as antimicrobial agents [64]. Colistin is a cationic peptide antibiotic, able to interact with anionic molecules, forming negatively charged nanoparticles for its delivery and shows a good activity on multidrug-resistant Gram-negative bacteria [65], including nosocomial pneumonia. The resulting polyelectrolyte complexes obtained mixing HA and colistin had good loading efficiencies and were characterized by modified release (45% and 85% of CT released in 15 and 60 min, respectively) compared to pure CT (100% CT released in 15 min).

## 3. Applications

Due to the wide range of biological and physical–chemical properties such as biodegradability, biocompatibility and viscoelastic properties, applications of HA have been explored in many fields. HA in its naturally-occurring linear form as well as in chemically modified formulations has several interesting medical, pharmaceutical, food and cosmetic uses as discussed below.

### 3.1. Hyaluronic Acid Solutions for Medical and Pharmaceutical Fields

(a) Ophthalmology

HA has been extensively investigated in the treatment of dry eye disease, of which sufferers experience visual disturbance and tear film instability. HA was shown to decrease surface stress, improve contrast sensitivity and optical surface quality. HA can lubricate the ocular surface reducing friction during blinking and ocular movements. Thus, the water retention and lubricant properties of HA are applied directly to the benefit of dry eye [66].

(b) Rheumatology

HA is naturally present in the synovial fluid, joint capsule and articular cartilage and has found a wide application in orthopedics, in particular, in osteoarthritis or rheumatoid arthritis. In fact, osteoarthritis is a disease, which can cause a substantial disability and decline in quality of life. In this context, HA is able to promote the synthesis of cartilage matrix, to prevent its degradation, and to reduce inflammation [67].

(c) Dermatology

HA can act as dermal fillers, anti-wrinkle agents and can promote tissue regeneration, improving skin quality [68].

Both the relatively rapid degradability of HA in living tissues and its low mechanical stability are often a challenge for the broad applicability of HA in the clinic as a versatile biomaterial. Moreover, the scarce elastic properties limit its direct use as filler because it is not able to lift tissues [69]. On the other hand, the possibility of easy chemical modifications of HA can allow to tune its physical and biological properties, such as solubility cohesivity and amphiphilicity.

In this view, an accurate definition of composition of HA derivatives is also mandatory to optimize the final properties of dermal fillers in terms of biocompatibility, biodegradability and mucoadhesivity [13].

### 3.2. Hyaluronic Acid as Porous Scaffolds and Three-Dimensional Models by Different Thechnologies

In the last decade, HA was variously used to fabricate three-dimensional (3D) scaffolds for various therapeutic applications (i.e., bone, space-filling, nerve and brain repair, cell delivery, muscle regeneration) [70,71]. The idea is based on fabricating only temporary supporting structures able to promote cell activities (i.e., adhesion, spreading, tissue ingrowth) thanks to the use of hydrogel-like materials with improved transport properties that promote cell growth, nutrient diffusion including biochemical/biophysical signals present in the native tissue [72]. However, it is mandatory to use processing technologies that allow the manipulating of HA by imparting controlled 3D architectures at micro- and/or sub micro- scale without altering their peculiar properties. Hence, a detailed focus on the use of major processing routes inspired by additive manufacturing approach—i.e., electro fluid dynamic, 3D bioprinting—was reported, mainly emphasizing the current advances related to the design and fabrication of HA-based 3D platforms for tissue engineering applications.

#### 3.2.1. Electro Fluid Dynamics

Electro Fluid Dynamics (EDTs) identifies a class of processes driven by electrostatic force interactions with polymers in solution, for the fabrication of fibres/particles with different shapes and sizes from micro to nanometric scale. The main advantage of these techniques in comparison with traditional processing approaches concerns the great opportunity to particularize materials properties down to the nano-scale, efficiently reproducing the peculiar organization of the ECM of natural tissues. Hence, an accurate setting of process conditions and experimental setups may be successfully used to design innovative platforms (i.e., scaffolds, porous materials, micro and nanoparticles) with tailored features as a function of the specific application demands. In this section, the focus was mainly addressed to the application of different processing modes (i.e., electrospinning, electro spraying, atomization) for the fabrication of HA-based devices.

(a) Electrospinning for micro to nanofibres

Electrospinning is the most popular technique for the fabrication of nanofibres due to its simplicity, cost–effectiveness, flexibility, scale-up potential and ability to spin a broad range of polymers [73]. The working principle of the electrospinning (ES) technique is based on the mechanical stretching of a viscoelastic solution to form fibres by the application of electrostatic forces generated via high voltage electric fields. Final morphological properties of the fibres can be optimized by controlling several factors—mainly related to solution properties, process and environmental conditions—that can affect the final size, morphology and properties of fibres [74]. From the operative point of view, a polymer solution is forced to be moved with a constant flow rate through a metallic channel with micrometric diameters, connected to a high voltage power supply. Once a high voltage is applied over a critical value—it overcomes the surface tension forces of solution at the needle tip, thus generating repulsive electrical forces. Consequently, a polymer jet is ejected, due to the polar interactions among polymer chains that provide a net mass transport from needle to the collector. During the travel between the tip and collector, solvent evaporation occurs, leaving the polymer fibres [75]. In this view, HA derivatives have a great potential for being utilized in a fibrous form via electrospinning technique for the fabrication of 3D scaffolds able to mimic the architecture and, to some extent, the chemical composition of the native ECM, also minimizing the effects of the inflammatory response that often characterizes the use of synthetic polymers in vivo [76,77].

However, experimental studies currently focused on HA-based nanofibres are very limited. This mainly depends on high viscosity levels, low evaporability and high surface tension of solutions, also in highly diluted conditions, which make it difficult to process them via electrospinning. Indeed, HA is a class of a highly water soluble natural polymers able to form highly polar aqueous solutions, characterized by high electrical conductivity and low volatility that may strongly against the mechanisms of polymer breaking due by the local interactions with high voltage electric field. Moreover, most parts of HA, such as other biopolymers, cannot be dissolved in organic solvents, typically characterized by low boiling points, that usually encourage the fibre formation mechanism driven by an efficient evaporation of the solvents. In a few other cases, HA derivatives could be dissolved in organic solvents with negative effects on their chemical stability (i.e., degradation, depolymerization) [78].

**Figure 6:**
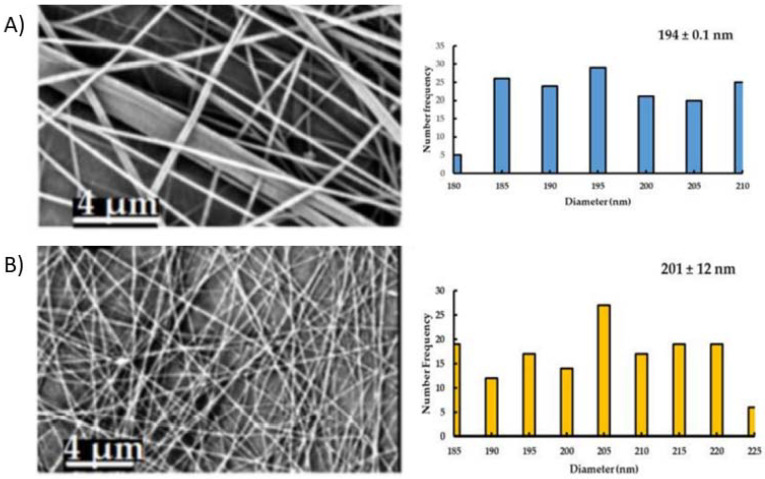
Electrospun fibres prepared by hyaluronic acid (HA) and Polyethylenoxide blend (**A**) and the addition of propolis (**B**): effect of the active compound on the morphology, distribution (e.g., number frequency) and average fibre diameter. Adapted with permission from [79].

In this view, an accurate definition of solution composition—by the sage coupling of additives, surfactants or other chemically modified species (Figure 6)—and a tailored setting of process parameters can be mandatory to reach the required standard in terms of feasibility and reproducibility for an efficient nanofibre production [79].

For instance, Junxing Li et al. have investigated the use of aqueous hyaluronan solutions via electrospinning, optimizing the distance between the needle and the collecting electrode in order to minimize the formation of irregular beads among fibres [80]. In order to overcome some problems of jet instability, they compare the behavior of two solutions using dimethyl DMF and distilled water (DW). Moreover, to improve the processability, gelatin as surfactant was also included to reduce the surface tension of the HA solution. Indeed, one of the most relevant issues to process HA via electrospinning is to reach the critical chain entanglement concentration that assures the topological conditions, i.e., formation of an adequate number of entanglements among chains, to regulate the local motion of polymer chains in solution [81]. However, this condition usually reached in the case of high solution viscosity, is hard to be obtained in the case of HA solution [82]. Hence, a common approach to improve the processability involves the blending of HA with other biopolymers working as uncharged carrier polymers, i.e., collagen [83,84] and zein [85]. Alternative approaches that avoid the use of other polymers, generally involve the implementation of modified electrospinning setups based on the use of more complex configurations (i.e., coaxial electrospinning [86] and/or additive driving forces such as temperature or assisted air flow (i.e., blow-assisted electrospinning) [87]. For instance, bilayer nonwoven membranes were recently prepared from chitosan (CS) and hyaluronic acid (HA) via needleless electrospinning [88]. In this case, HA composing the second layer did not require any crosslinking treatment, being able to strictly penetrate into the pores of the surrounding CS layer, due to the strong ion group interactions among the polymer chains. As a consequence, the tendency of HA to rapidly disappear in in vitro culture is significantly reduced, without any negative effects on cell biocompatibility.

(b) Electrospraying for nanoparticles and microgels

**Figure 7:**
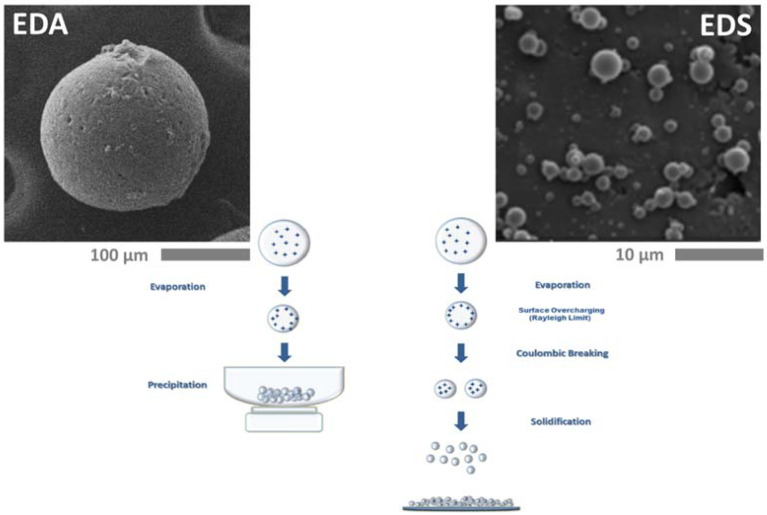
Representative scheme of electro fluid dynamics (EDT) processing modes: Electro Dynamic Spraying (EDS) vs. Electro Dynamic Atomization (EDA). Adapted from [98]. Copyright (2016), with permission from Elsevier.

Electrospraying is a versatile EDT mode to produce micro and nanoparticles by the interaction of the polymer solution with a high voltage electric field. In this case, the interaction of injected charges of liquid and external electric field originates the breaking off the solution into a homogeneous population of fine droplets, due to a balance of repulsive forces generated by the surface charges exceeding surface tension [89]. Indeed, during the process, charges tend to aggregate onto the liquid surface so forming unstable surfaces that provide the atomization of the polymer solution into smaller droplets—from the micro-scale to nano-scale—only by electrical forces generated by the Coulomb such as charge interactions, without the administration of additional mechanical energy [90]. As a function of the mechanism of solvent removal, two different processing modes can be distinguished (Figure 7):

I. Pure Electro Dynamic Spraying (EDS): the fabrication of nanoparticles is driven by the fast evaporation of volatile solvents that promotes strong repulsive charges interactions by the confinement and aggregation of charges into nanometric droplets [91,92].
II. Microdropping or Electro Dynamic Atomization (EDA): the fabrication of microsized particles is determined by the extraction of low volatile solvents via thermodynamic routes (i.e., solvent/non solvent extraction) or by chemical crosslinking or physical gelation (Figure 7) [93,94].

Both techniques have been recently investigated for the fabrication of HA-based particles for molecular release [95] confirming the promising use of HAs as carrier in drug delivery, targeting and screening strategies for personalized medicine [13]. Among them, ES technique, initially proposed an alternative technological solution to the use of traditional emulsion, is also successfully emerging in integrated approaches for fabrication of more complex constructs that combine nanoparticles and electrospun fibre network (i.e., additive electrospraying [96] suitable to “separately” controls release and functional properties of 3D scaffolds for tissue engineering [97]. Otherwise, EDA is successfully applying to non-Newtonian polymers solutions such as polysaccharides (i.e., HA, alginate, cellulose or chitosan) to produce cell loaded microgels to investigate cell and molecular activities in health or pathological niche [98] as alternative to consolidated microfluidic approaches [99,100].

#### 3.2.2. Three-Dimensional Printing

**Figure 8:**
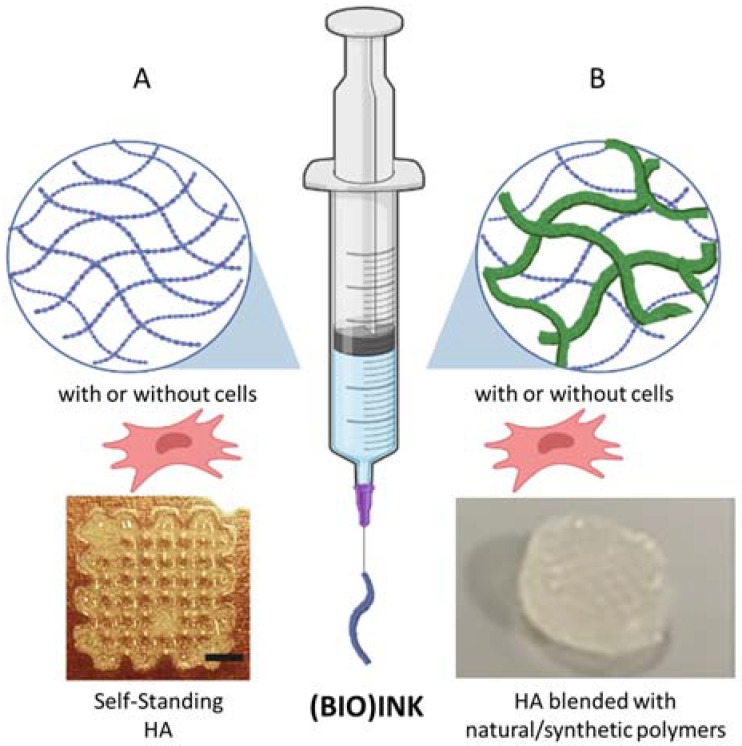
Schematic representation of hyaluronic acid (HA)-based (bio)ink as (**A**) self-standing material and (**B**) blended with other natural/synthetic polymers. Example of 3D printed: (**A**) methacrylated HA (3% (*w*/*v*)). Adapted with permission from ref. [107]. (**B**) HA (1% (*w*/*v*)) blended with alginate (2% (*w*/*v*) into a polylactic acid structure. Adapted with permission from [108].

Among the biomaterials, HA-based hydrogel and its derivatives stand out, in additive manufacturing, for their cytocompatibility, their ability to hold living cells, their tunable mechanical/rheological and biodegradation properties, and the possibility to obtain a good resolution during 3D printing [101]. Indeed, over the last few years, this technique and its evolution, 3D biofabrication, have emerged as a powerful tool for the computer-assisted fabrication of patient specific scaffolds for tissue engineering and regenerative medicine. By using a layer-by-layer approach, these techniques may allow the production of complex biologic products, with high precision and control over physical–chemical and morphological parameters, from raw materials such as polymeric biomaterials, growth factors (GFs) and/or the combination of living cells [102]. In this scenario, the right selection of the biomaterial and its appropriate strategy of functionalization, as well as the correct choice of 3D printing technique have boosted great advances in material science and engineering. In general, 3D printing includes various techniques, which differ for the specific working principle: extrusion, inkjet, laser jet and stereolithography (SLA). However, one of the most important components of all these techniques is the employed ink, in the form of a liquid hydrogel, which has to preserve its shape before and after the printing (Figure 8) [102,103,104]. Herein, throughout this paragraph, when referring to the ink and fabrication, with or without living cells, the following general nomenclature will be used: (bio)ink and (bio)fabrication. Extrusion-based (bio)printing is a cheap and simple technology, which uses a mechanical screw to extrude a wide range of biomaterials. Inkjet (bio)printing is based on a non-contact low viscosity ink deposition technology, which can be thermally, piezoelectrically or electromagnetically controlled. Laser-assisted (bio)printing employs a laser as energy source to deposit the (bio)ink, which may have a high viscosity. Finally, SLA technology consists of a liquid material, which can be photocured by ultraviolet (UV) light. One the main limitations of this technique is the difficulty in achieving a large-scale production. There are other methods such as acoustic, microwave, electro-hydrodynamic and volumetric (bio)printing, which are, actually, under investigation for the design of tissues and organs [101,105,106]. Currently, HA and its derivatives, as self-standing materials or combined with other natural or synthetic polymers, have been mainly processed by extrusion-based and SLA techniques (Figure 8) [107,108].

(a) Three-dimensional printing of neat self-standing HA

The rheological behavior of neat HA, with its clear prevalence of viscous modulus, has limited its applicability in 3D printing. Indeed, HA (bio)inks are characterized by poor printing properties. To date, a few works show the use of neat HA or its functional derivatives, even though the design of innovative HA (bio)inks for 3D printing, without blending with other polymers, has been receiving increasing attention. Liu et al. tried to optimize the printability of neat HA, even without adding functional groups. In particular, the authors explained the mechanisms of dynamic coordination and the interactions among the parameters, which can fine tune the crosslinking and the phase transition. Accordingly, they selected the suitable conditions to 3D print HA using cold-stage or direct writing approaches. Particularly, they demonstrated that the concentrations of Fe^3+^ and H^+^ ions and the reaction time allowed the tunable ratios of mono-, bi-, and tridentate coordination, leading to low-to-high crosslinking densities and reversible solid-liquid phase transition of the hydrogels [109]. However, chemical modifications of HA remain the most employed strategy to endow a shear thinning behavior in order to improve the printability of the (bio)ink formulations. For example, adamantane-modified HA and a beta-cyclodextrin-modified HA were developed by Burdick et al. The interactions between the two hydrogels were based on a guest– host non-covalent and reversible bond. The guest-host network generated a shear-thinning and rapidly self-healing hydrogel [110,111,112]. Furthermore, Petta et al. described the development and the optimization of a tyramine-modified hyaluronic acid (HATyr) bioink based on a dual crosslinking system, particularly, an enzymatic crosslinking mediated by horseradish peroxidase (HRP) and hydrogen peroxide (H_2_O_2_), followed by a visible light crosslinking triggered by Eosin Y. The authors showed the possibility to tune the viscoelastic properties of the enzymatically crosslinked HA-Tyr. Afterwards, by exposing the bioink to green light during the printing process, 3D constructs encapsulating viable cells for up to two weeks were fabricated [113,114,115]. Pentenoate-functionalized HA hydrogels (PHA), with different M_w_, were developed as printable bioink. The authors found a yield stress of ∼1 kPa for PHA and storage modulus recoveries above ∼85%. Based on those results, they selected PHA (M_w_ = 1.5 × 10^6^) at 4 wt% and PHA (M_w_ = 1 × 10^6^) at 8 wt%, which showed improved printability. From a biological point of view, they showed that by increasing cell density in PHA up to 9 × 10^6^ cells/mL, a minimal effect on the printability was detected [116]. Maleiated sodium HA (MHA) with a high substituted degree of acrylate groups and thiolated sodium HA (SHHA) were synthesized by Wan et al. [117]. The results showed that 3D-printed well-defined MHA/SHHA scaffolds exhibited a fast-gelling ability, improved mechanical properties in compression of scaffolds and cytocompatibility [117]. HA functionalized through the reaction with glycidyl methacrylate or methacrylic anhydride (HAMA) was used as (bio)ink to print scaffolds, which showed enhanced vascularization and host tissue ingrowth, with a limited inflammatory response [118]. HAMA (bio)ink was also tested for the manufacturing of scaffolds for bone tissue engineering and advanced smart 3D-printed patches for wound healing and as a delivery platform of exosomes derived from human mesenchymal stem cells (hMSC-EXOs) [107,119,120,121,122,123]. The 3D-printed HAMA patches showed in-creased mechanical performances, appropriate swelling ratio, longer residence time, and suitable biocompatibility. Furthermore, HAMA-hMSC-EXOs improved the biological behavior of human fibroblasts and human endothelial cells in terms of proliferation, migration, angiogenic ability, and expression of specific markers related to wound healing [119,120].

(b) Three-dimensional printing of HA combined with natural/synthetic polymers

Over the last years, HA has been mainly employed in combination with other synthetic, mainly polylactic acid (PLA), polycaprolactone, PEG, and natural polymers such as gelatin, collagen, gellun-gum, nanocellulose and their derivatives. Indeed, the blending or the use of a support matrix can lead to an improvement in the viscosity of the ink formulation, the final mechanical stability or the biological properties [103]. A thixotropic bioink made from methacrylated collagen I, thiolated HA, and gelatin nanoparticles was processed to produce tubular structures. This bioink was also able to support human liver cancer cell line (HepG2) in bioprinted organoids [124]. HA, hydroxyethyl acrylate (HEA) and methacrylated gelatin (GelMA) were used as bioink with bone cells. The authors synthesized the hydro-gel by graft polymerization of HEA to HA and then grafting of GelMA via radical polymerization. They showed the tunability of physico-chemical and biological properties of these hydrogels by varying the concentration of methacrylic anhydride in the gelatin modification [125]. An injectable hydrogel composed of HA-tyramine (HATA) and gelatin-hydroxyphenyl propionic acid (GH) was developed via oxidative coupling reaction using HRP and H_2_O_2_. The gelation rate and mechanical properties of the hydrogel were controlled by modulating HRP and H_2_O_2_ concentrations. The blending with GH allowed improving stability and cellular behaviors. In addition, the injectable hydrogel showed good printability with high cell viability after one day of printing [126]. GelMA/gellan-gum/HAMA were used to investigate the chondrogenesis process of articular cartilage progenitor cells (ACPCs) and multipotent MSCs embedded in these bioinks. Two-zone constructs were printed with GelMA/gellan-gum/HAMA containing ACPCs in the outer region and MSCs in the inner one. The inclusion of HAMA increased filament stability but, surprisingly, no significant differences in matrix production or gene expression by chondrocytes, ACPCs or MSCs were observed [127]. Scaffolds of collagen with HA were fabricated using 3D printing. The dehydrothermal crosslinking treatment improved resistance to collagenase and mechanical properties. Furthermore, the inclusion of HA showed higher resistance solubilization than scaffolds without treatment in a short time at physiological conditions [128]. Collagen-I (3 mg/mL) was also used with HAMA (10 mg/mL) as bioink with the neural GF and glial cell line-derived neurotrophic factor. The bioink was bioprintable, improved cell viability compared to molded controls, and was conducive for cell adhesion, GF sequestration and neural cell infiltration [129]. HA linked N-(2-aminoethyl)-4-(4-(hydroxymethyl)-2-methoxy-5-nitrosophenoxy) butanamide (NB) was combined with GelMA to print functional living skin using a digital light processing (DLP)-based 3D printing technology. The bioink demonstrated its rapid gelation kinetics, tunable mechanical properties, good biocompatibility and tissue adhesion. In vivo study showed that the material was able to promote dermal regeneration in large animals [130]. In the field of skin tissue regeneration, catechol-HA (HA-CA), alginate and gelatin were printed to develop a useful platform. Indeed, the printed scaffolds demonstrated high elasticity and supported the formation of a double-layered cell-laden skin-like structure [131]. Successively, adipose-derived stem cells (ADSCs) were combined with functionalized HA and sodium alginate hydrogels for cartilage tissue engineering by Nedunchezian et al. In particular, biotinylated HA was synthesized via an ADH linker with amide bond formation to form HA-biotin (HAB). A partially crosslinked HA-biotin-streptavidin (HBS) hydrogel, mixed with sodium alginate, was printed and physically crosslinked in calcium chloride. The results demonstrated that the functionalization with streptavidin positively influenced the printability as well as the structural integrity of scaffolds, which showed a well-defined geometry, as designed, and a favorable biocompatibility profile. Furthermore, they allowed the increasing of the expression of chondrogenic marker genes and sulfated GAGs [132]. Bioinks composed of decellularized human heart ECM (dhECM) with either GelMA or GelMA/HAMA hydrogels, dual crosslinked with UV light and microbial transglutaminase (mTGase), were also developed for cardiac tissue engineering. The hydrogels showing improved mechanical properties were extrudable and compatible with human-induced pluripotent stem cell derived cardiomyocytes (iCMs) and human cardiac fibroblasts (hCFs). Tissue-like beating of the printed constructs with striated sarcomeric alpha-actinin and connexin 43 expression was observed [133]. Lee et al. developed a method to synthesize a bioink using HA (up to 30% (*w*/*v*)) and sodium alginate without employing the chemical crosslinking agents of HA. The bioink also encapsulated NIH3T3 fibroblast cell line. The results showed that the blended hydrogel was successfully printed with viscosi-ties ranging from 883 Pa*s (HA, 0% *w*/*v*) to 1525 Pa*s, (HA, 30% *w*/*v*) at a 0.1 s-1 shear rate. The relative proliferation rate of the encapsulated cells in the blended bioink was 70% higher than the sodium alginate bioink after the fourth day [134]. Biopolymers such as nanofibrillated cellulose or its derivative, carboxymethylcellulose (CMC), have showed excellent characteristics for use as bioinks due to their excellent biocompatibility and rheological properties. For example, 3D printability of HA/CMC hydrogels crosslinked through N-acyl-hydrazone bonding was studied by Janarthanan et al. The shear thinning and self-healing properties of the hydrogel allowed printing 3D constructs with different geometries up to 50 layers with high precision and high post-printing stability. In vitro cytotoxicity studies confirmed the excellent cytocompatibility of these gels. In vivo mice studies proved that these biocompatible hydrogels enhanced angiogenesis [135]. Three-dimensional nanocellulose/alginate/HA scaffolds embedding D1 mesenchymal stromal cells (D1-MSCs) were bioprinted and widely characterized embedded for cell viability analysis. The addition of HA highlighted the possibility to improve scaffold properties, in terms of higher biocompatibility and cell viability in comparison with the scaffolds without HA [136]. Recently, hydrogels have been also combined with graphene oxide (GO) and bioactive glasses (BG), due to their angiogenic properties. GO/HA/chitosan composite hydrogel with different mass ratios of GO (from 0.1% to 1%) were processed to obtain stable composite scaffold through 3D printing technology. Multilayered scaffolds with an interconnected and open porosity were successfully obtained. Furthermore, the porosity gradually decreased (from 94 to 40%) by increasing GO concentration. Meanwhile, water swelling rate, degradation rate and elastic modulus improved. In terms of biological behavior, 0.25% GO concentration allowed promoting cell growth and proliferation [137]. In the work of Bertuola et al., the authors studied the printability and the rheological properties of gelatin/alginate/HA inks with 2–8% wt of 45S5 BG. The storage modulus of the inks decreased by increasing the BG concentration indicating that the microparticles might negatively influence the polymeric network. Furthermore, a reduction in the viscosity was determined at BG concentrations above 6%. Inks without BG or up to 2% evidenced the best printing fidelity. The tensile modulus of crosslinked 40%-filled scaffolds increased from 130 kPa (without BG) to 160 kPa (6–8% BG). From the biological point of view, the inks were cytocompatible and a hydroxyapatite layer was detected in scaffolds containing BG 6% and 8% wt after being cultured for 2 days [138]. Successively, a hydrogel bioink based on alginate and HA crosslinked through multiple crosslinking mechanisms, i.e., acyl-hydrazone, hydrazide interactions and calcium ions, has been designed by Janarthanan et al. The gels showed a shear thinning behavior with tunable mechanical properties. Among the different combinations of gels compositions prepared, the A5H5 (Alginate-acyl-hydrazide:HA-monoaldehyde, ratio 50:50) gel showed a gelation time of ∼60 s, viscosity of ∼400 Pa*s (at zero shear rate) and an increased degradation time higher than 50 days. The A5H5 gel showed high printability with increased post-printing stability. Furthermore, scaffolds showed a well-defined interconnected structure with high stability in various pH solutions and increased mechanical properties. In vitro assays confirmed the cytocompatibility of these gels. In in vivo studies, Alg-HA gel showed high biocompatibility (>90%) and increased angiogenesis and reduced macrophage infiltration, demonstrating the promising potential of these hydrogels in 3D bioprinting applications for tissue engineering and regenerative medicine [139]. Based on dynamic hydrazone-crosslinked HA (HA-HYD) and photocrosslinked GelMA, a double-network (DN) hydrogel with significantly enhanced mechanical strength, self-healing, and shear thinning properties was developed as a printable hydrogel bioink. Owing to shear thinning, the DN hydrogel bioinks could be extruded to form uniform filaments. The self-healing performance of the filaments and photocrosslinking of GelMA allowed the production of a stable printed structure with high mechanical strength. The in vitro cytocompatibility assay showed that the DN hydrogel printed scaffolds supported the survival and proliferation of BMSCs [140]. Recently, Hauptstein et al. designed a dual-stage crosslinked HA-based bioink that enabled covalent tethering of transforming GF-beta 1 (TGF-β1). The bioink was made by 3,3′-dithiobis(propanoic dihydrazide), thiolated HA, PEG-diamine, and PEG-diallyl carbamate. BMSCs were cultured over three weeks in vitro. Cells within the constructs with tethered TGF-β1 are able to differentiate in chondrocytes, if compared to not functionalized scaffolds. Furthermore, 3D printing did not affect the functionality of covalently tethered TGF-β1 [141]. ECM has also been used to produce printable bioinks. In particular, it was combined with HAMA, containing visible light initiators, and used for extrusion-based and DLP-based bioprinting. The results showed that the mechanical properties of HAMA hydrogel significantly improved by addition of 10 mg/mL of ECM. The compressive strength and modulus increased 2.7 and 3.1 times more than those of neat hydrogel, respectively. The cell culture test showed excellent biocompatibility [142].

Even if a totally natural polymer (bio)ink has been always preferred, the use of synthetic polymers as support matrix is gaining interest, especially in the design and realization of load-bearing structures with high mechanical requirements. For example, in the field of articular cartilage regeneration, HA-based bioink (1% (*w*/*v*)) was mixed with Alginate (2% (*w*/*v*)) and used as cell-carrier biomaterial. In particular, it was printed with chondrocytes into the pores of a PLA structure, previously produced. The bioink improved cell functionality by an increase in the expression of chondrogenic gene markers and specific matrix deposition and, therefore, tissue formation [91]. Hauptstein et al. used thiolated HA, allyl-modified poly(glycidol) and 1 wt% unmodified high M_w_ HA with a PCL support matrix. Results showed improved ECM distribution in those constructs [143]. The combined use of 3D-printed PLA and HA was shown also a potential tool to enhance bone augmentation by Yun et al. [144].

Collectively, all the presented HA and HA-based formulation (bio)inks have enabled the generation of high-quality tissue engineered scaffolds for a wide range of applications: bone, cartilage, cardiac tissue engineering, among others. However, research is ongoing and other innovative HA-bioinks, suitably decorated with suitable GFs, are still requested to improve the specific tissue repairing process. Furthermore, it is worth noting that 3D printing is evolving towards 4D printing, in which the material should be able to adjust its shape, properties and functionalities according to external stimuli such as temperature, pH, light, electric or magnetic fields. For this reason, the development of HA-based self-standing (bio)inks with optimized crosslinking density, tunable mechanical behavior, biocompatibility and other properties such as self-healing, in situ gelation and shape memory are still a significant challenge.

## 4. Conclusions and Future Perspectives

During the last decade, HA has found an incredibly high number of commercial applications in medicine and in cosmetics as eye drops, skin creams, in products to treat gastroesophageal reflux, in medical devices and material engineering, even in beverage and soft drinks, in soaps and so on. The natural linear polysaccharide HA is a biomaterial that exhibits good water absorption in the form of hydrogels and allows maintenance of tissue hydration, which facilitates cell infiltration. The possibility of chemical and physical modification of HA has allowed the development of drug delivery systems and new materials with new properties. However, it is necessary to further improve the current crosslinking methods of HA-based hydrogels to ensure that the resulting structures have good biocompatibility, stability, sustainability, as well as suitable printability, degradation time, mechanical properties and other relevant aspects for the development of more effective and tailor-made materials. Further improvements in nanotechnologies and materials science will be essential to design novel HA-based hydrogels, by the synthesis/modification of new formulations of biomaterials, and the implementation of new experimental setup configuration suitable to better mimic the complexity of natural tissue and to accelerate the related product development for clinical translation.

## Figures and Tables

**Table 1:** Commercially used crosslinkers for HA’s hydroxyl group modifications.

Name	Chemical Structure	References
1,4-Butanediol diglycidyl ether	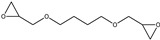	[20,21,22]
Poly(ethylene glycol) diglycidyl ether	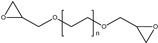	[20,21,22]
1,2,7,8-Diepoxyoctane	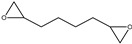	[22]
Divinylsulfone	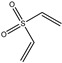	[22,23]
Octenylsuccinic anhydride	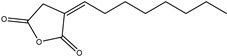	[24]
Methacrylic anhydride	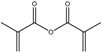	[25]
Glutaraldehyde	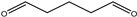	[26,27]

**Table 2:** Commercially used crosslinkers for HA’s carboxyl groups modifications.

Name	Chemical Structure	References
Diamines	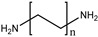	[14,29]
Biscarbodiimides	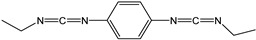	[22,30]
